# Biomass potential of novel interspecific and intergeneric hybrids of *Saccharum* grown in sub-tropical climates

**DOI:** 10.1038/s41598-020-78329-8

**Published:** 2020-12-09

**Authors:** Mintu Ram Meena, Ravinder Kumar, Karuppaiyan Ramaiyan, Manohar Lal Chhabra, Arun Kumar Raja, Mohanraj Krishnasamy, Neeraj Kulshreshtha, Shashi Kant Pandey, Bakshi Ram

**Affiliations:** 1 ICAR-Sugarcane Breeding Institute, Regional Centre, Karnal, India; 2 ICAR-Sugarcane Breeding Institute, Coimbatore, India

**Keywords:** Physiology, Plant sciences

## Abstract

Sugarcane-derived biomass is a promising source of renewable energy to meet the growing demands for biofuel. Currently, modern sugarcane cultivars are unable to provide enough biomass due to their narrow genetic base and susceptibility to abiotic and biotic stresses. We have evaluated total of 23 hybrids derived from diverse genetic backgrounds of different *Saccharum* spp. and allied genera, one inbred and compared with commercial checks. Intergeneric hybrids (IGHs) KGS 99-100 and GU 04-432, produced significantly higher biomass (43.37 t ha^−1^ and 35.24 t ha^−1^, respectively) than commercial sugarcane have genes derived from *Erianthus arundinaceus*. Interspecific hybrids (ISHs) GU 07-3704 and 99-489, also produced significantly higher amounts of biomass (37.24 t ha^−1^ and 33.25 t ha^−1^, respectively) than commercial checks have genes from *S. officinarum* and *S. spontaneum* backgrounds. ISHs recorded significantly higher biomass yield, number of stalks and total dry matter percentage whereas, IGH group recorded significantly higher fibre percent. Furthermore, the clones resistant to red rot and sugarcane borers were identified. The estimated energy value for seven hybrid clones was found to be very high. Cluster analysis of genetic traits revealed two major clusters in traits improving biomass. Our study has revealed that the genetic diversity present in these hybrids could be used for improving biomass production and tolerance to abiotic and biotic stresses in cultivated sugarcanes.

## Introduction

The substantial use of fossil fuels in the twenty-first century has resulted in a huge upsurge in atmospheric CO_2_ levels leading to global warming and other environmental effects. In addition, sudden surges in crude oil prices in the international market are driving economies to develop new sources of environmentally friendly and renewable energy sources. In a recent review, Fawzy et al. (2020)^1^ have lately evaluated several mitigation strategies to reduce CO_2_ emissions by using renewable energy sources, fuel switching, efficiency gains, nuclear power, and carbon capture storage and utilization methods. Amongst these strategies, plant biomass-derived biofuels hold great potential as replacements for fossil fuels to reduce greenhouse gas emissions.

Sugarcane (*Saccharum* spp.) is an important industrial crop grown in over 80 tropical and sub-tropical countries. Globally, ~ 26.9 million hectares of land are used for sugarcane cultivation; these produce 1.9 billion tonnes of fresh sugarcane, roughly translating to a yield of ~ 70.9 tonnes/hectare^2^. Sugarcane is a C4 plant and so has a very efficient photosynthesis process and an exceptionally high biomass accumulating capacity in the form of carbohydrates; it also has a much higher energy input/output ratio than most other crops^3,4^. Sugarcane-based biomass, therefore, can be a low-cost energy production system^5^ and a versatile source of renewable energy as ratooning allows the growth and harvest of four or five crops without replanting^6^.

One of the major limitations in using plant biomass for combustion/pyrolysis in energy production, is the production of toxic nitrogen oxides; nonetheless, a study by Osman (2020)^7^ has demonstrated that coupling the DeNOx catalyst with urea to construct an *in-situ* selective catalytic reduction (SCR) NH_3_-SCR system during the combustion process can significantly reduce nitrogen oxide emissions.

In order to reduce greenhouse gases (GHGs) emissions, large scale deployments of negative emissions technologies (NETs; i.e. technologies that result in the net removal of CO_2_/GHGs from the atmosphere) are required^8^. The major forms of NET currently in use include technologies for bioenergy carbon capture and storage, and biochar and soil carbon sequestration^1^. Most of these technologies, however, are currently only in the demonstration stages, and large-scale efforts are still required to offset the current levels of CO_2_ emissions produced by humans. Since sugarcane is a high biomass producing crop, it could be used to convert CO_2_ into soil carbon; one study shows that sugarcane plantations can convert more CO_2_ into soil carbon than matured or secondary forests^9^. It has been found that sugarcane yields in Australia increased by 8% and 4% with weak climate change and moderate climate change, respectively, but were reduced by 10% with strong climate change^10^. Sugarcane based biomass (bioenergy) is a low cost energy production system^5^ and a versatile source of renewable energy as it allows four to five times harvest of ratoon without replanting^6^.

To date, sugarcane is among the most efficient crops in the world along with other C_4_ grasses when it comes to conversion of solar energy into stored chemical energy and biomass accumulation^11^. It has been estimated that more than 700 Mha of land being used to grow cereal crops produced a total of only 2400 mt year^−1^ of biomass, whereas only 21 Mha of sugarcane crops alone produced 1750 mt year^−1^ of biomass^12^. Therefore, sugarcane may provide a vital solution to many environmental problems without reducing economic benefits at the global level.

Conventionally, in the sugar industry, the “bagasse” that remains after juice extraction through crushing is an important by-product used to generate electricity and produce fertilizers^13^. More recently, it has been used for biofuel production^14,15^. However, the production cost for bioconversion of sugarcane biomass into biofuel is still considered to be relatively high, which makes it difficult to commercialize on a large scale^16^. Therefore, it is essential to develop improved sugarcane varieties with high biomass yield and fibre content, which also have better biomass degradability; in addition, we also need better enzyme digestion technologies to maximize the efficiency of conversion of sugarcane biomass into biofuel^17^.

Brazil is the first country to have successfully launched an ethanol-based fuel program (ProAlcooL), which produces alcohol on a large scale from sugarcane^18^; in 2014, Brazil produced around 23.4 billion litres of ethanol from sugarcane biomass (Renewable Fuel Association, 2015). However, India has also successfully exploited the energy potential of sugarcane to produce ethanol and electricity^19^. To enhance Indian’s energy security, the Government of India (GoI) aimed to increase ethanol blending percentages in gasoline fuels from 5 to 20% by 2017.

Since usage of biofuels now plays a significant role in drafting the energy policies of many countries, several studies have shown that producing biomass for use as biofuels can be significantly increased^20^. Unfortunately, at present, the cane varieties cultivated in India have only 13–15% fibre, and the development of high biomass producing cultivars has enormous potential to meet the energy demand in the country^21^.

In India, sugarcane is grown under both tropical and subtropical areas. The sub-tropical region faces climatic vagaries throughout the year, such as flooding, extreme winter and summer temperatures, drought, and salinity, all of which are major constraints for crop production. Therefore, in order to be able to grow high biomass producing sugarcane crops under subtropical regions, we need to develop high biomass-type clones which are also tolerant to several different types of environmental stresses^22^.

There is tremendous genetic variation found within the *Saccharum* species and allied genera of *Miscanthus,* and *Erianthus* species that can be exploited for high biomass and fibre in sugarcane breeding programs^23^. However, in the recent past, although the genetic diversity of *S. officinarum* has been exploited towards this end, the genetic diversities of *S. spontaneum*^24^, *S. robustum* and other allied genera have not been explored^25^.

The ICAR-Sugarcane Breeding Institute, Coimbatore, has been working to generate intergeneric (IGH) and interspecific (ISH) hybrids of sugarcane with high biomass production potential using *S. spontaneum*, *S. robustum*, *S. barberi*, and *Erianthus arundinaceus*^26^. In this study, we evaluate the potential of IGHs and ISHs and we have generated as potential energy and fuel producers.

## Results

A high degree of variation among the clones under study (Table 1) for all the traits in both the years was observed. The interaction term for year and clone (year × clone) identity was significant for all traits except numbers of millable canes (NMC), which indicates that the performance of the clones varied between the two years of growth. Further, it was assumed that the interaction term genotypes x environment (G × E) plays a significant role in the performance of these hybrids over several years. However, the interaction between year and replication was found to be non-significant, indicating that replications over different years do not have a significant impact on the traits studied. The results of the two-way analysis of variance (ANOVA) for the eight biomass related traits for two years are shown in Table 2.

**Table 1:** Parentage of hybrid clones used in this study (S. no 1 to 17 are ISH clones, S.nos. 18 to 23 are IGH clones, 24 to 27 are commercial checks and, S. no. 28 is an inbred clone).

S.No	Clone name	Parentage (*S. officinarum, S. spontaneum, and S. robustum*)	Species	
			Female	Male
1	GU 092-410	IND 90-776 X Co 775	*S. spontaneum*	Commercial cane
2	99-81	Co 85002 X (PIO 88-96 x SIP-54)	Commercial cane	*S. officinarum*
3	99-132	Co 86002 x (PIO 88-1715 X IND-82-319)	Commercial cane	*S. officinarum* *S. spontaneum*
4	99-438	PIO 88-1809 X (PIO 88-100 x SIP-93-8)	*S. officinarum*	*S. officinarum*
5	99-488	PIO 88-110 X ( PIO 90-202 x SIP 32)	*S. officinarum*	*S. officinarum*
6	99-489	PIO 88-110 X (PIO 90-202 x SIP 32)	*S. officinarum*	*S. officinarum*
7	KGS 2004-13	Co 86002 X 97-244 (Pathri x Co 87268)	Commercial cane	*S. barberi and S. sinense*
8	KGS 2004-48	97-121 (Kansor x BC 82-175) X Co 90018	*S. barberi and S. sinense*	Commercial cane
9	KGS 2004-60	97-121 (Kansor x BC 82-175) X Co 775	*S. barberi and S. sinense*	Commercial cane
10	KGS 2004-72	97-121 (Kansor x BC 82-175) X Co 775	*S. barberi and S. sinense*	Commercial cane
11	KGS 2004-90	Co 86002 X 97–244 ( Pathri x Co 87268)	Commercial cane	*S. barberi and S. sinense*
12	KGS 2004-186	Co 88028 X 97-130 (Kansor x BC 82-175)	Commercial cane	*S. barberi and S. sinense*
13	GU 07-3704	PIO-88-110 X IND 00-1061	*S. officinarum*	*S. spontaneum*
14	GU 07-3730	PIO-88-110 X IND 00-1061	*S. officinarum*	*S. spontaneum*
15	GU 07-3764	PIO-88-110 X IND 00-1061	*S. officinarum*	*S. spontaneum*
16	GU 07-3784	PIO-88-110 X IND 00-1061	*S. officinarum*	*S. spontaneum*
17	GU 07-3849	PIO-88-1703 X IND 00-1058	*S. officinarum*	*S. spontaneum*
18	GU 98-1395	CoC 671 X IG 91-1100	Commercial cane	*Erinathus arundinaceus*
19	KGS 99-100	Co 7201 X IK 76-76	Commercial cane	*Erinathus arundinaceus*
20	KGS 99-104	Co 7201 X IK 76-76	Commercial cane	*Erinathus arundinaceus*
21	KGS 99-109	Co 7201 X IK 76-76	Commercial cane	*Erinathus arundinaceus*
22	GU 04-431	PIR 98-635 X IK 76-91	*S. robustum*	*Erinathus arundinaceus*
23	GU 04-432	PIR 98-635 X IK 76-91	*S. robustum*	*Erinathus arundinaceus*
24	Co 0238	CoLk 8201 X Co 775	Commercial cane	Commercial cane
25	CoJ 64	Co 976 X Co 617	Commercial cane	Commercial cane
26	CoS 767	Co 419 X Co 319	Commercial cane	Commercial cane
27	CoS 8436	MS 68/47 X Co 1148	Commercial cane	Commercial cane
28	1148-54-242-2	Co 1148 (Selfing)	Commercial cane

**Table 2:** Results of the two-way analysis (F-ratio) showing the effects of genotypes and year on the different traits of sugarcane clones (SCW: single cane weight, NMC: number of millable canes, TDM: total dry matter, DM: dry matter, FB: fresh biomass).

Source	F-ratio and significance								Fibre%	DBM
	Df	SCW	Cane brix	Juice brix	NMC	TDM	DM	FB		
Rep	1	3.666 ns	0.315 ns	3.202 ns	0.032 ns	1.258 ns	9.567**	0.000 ns	11.86**	9.54**
Year	1	21.159***	0.306 ns	26.328***	0.802 ns	189.649***	10.755**	49.918***	0.00	10.87**
Year*Rep	1	0.466 ns	2.816 ns	0.192 ns	0.011 ns	1.243 ns	0.294 ns	0.722 ns	1.166	0.318 ns
Clone	27	85.867***	37.955 ns	8.856***	34.439***	15.123***	52.531***	91.942***	10.95***	52.70***
Year*Clone	27	2.492**	0.200***	4.031***	0.339 ns	11.273***	2.379**	3.019***	7.06***	2.38**
Error	54									
C. Total	111									

Level of significance: ***, **, * and ns indicate significance at P < 0.001, P < 0.01, P < 0.05, and non-significance, respectively.

### Biomass production and traits affecting biomass production

**Figure 1:**
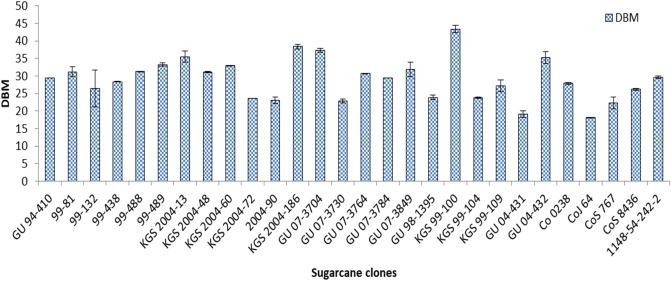
Dry biomass potential (DBM (t/ha)) of interspecific (ISH), intergeneric (IGH), and commercial hybrids of sugarcane.

The characteristics of ISHs and IGHs were compared with either commercial sugarcane or population means to identify clones with better-performing traits in biomass production. Out of the 28 clones evaluated, five ISHs and two IGHs recorded significantly higher dry biomass yields as compared to the population mean of 29.03 t ha^−1^. Eight ISHs—2004-186 (38.34 t ha^−1^), GU 07-3704 (37.24 t ha^−1^), KGS 2004-13 (35.43 t ha^−1^), 99-489 (33.25 t ha^−1^), KGS 2004-60 (32.93 t ha^−1^), GU 07-3849 (31.83 t ha^−1^), 99-488 (31.25 t ha^−1^), and 99-81 (31.15 t ha^−1^)—and two IGHs, namely, KGS 99-100 (43.37 t ha^−1^) and GU 04-432 (35.24 t ha^−1^) were significantly superior to the best commercial standard, Co 0238 (27.88 t ha^−1^), in dry biomass yields (Fig. 1). The estimated value of dry biomass produced ranged from 18.07–43.37 t ha^−1^ for all the studied clones. The lowest dry biomass yield (20.76 t ha^−1^) was recorded by the clone GU 04-431. The overall means for dry biomass yield for all ISHs and IGHs were 30.37 t ha^−1^and 28.75 t ha^−1^, respectively, while that of commercial varieties was 24.81 t ha^−1^^27^. The highest dry biomass yielding clone was KGS 99-100 (43.37 t ha^−1^), a hybrid clone between a commercial cultivar and *E. arundinaceus.*

Total dry matter (DM) accumulation ranged from 19.78% (GU 04-432) to 40.42% (GU07-3730). Four of the ISHs—GU 07-3730 (40.42%), GU 07-3784 (37.35%), KGS 2004-60 (35.80%), and GU07-3764 (32.53%)—accumulated significantly higher percentages of DM as compared to commercial cultivars at the time of harvest^28^. In energy cane, dry biomass yield is of primary importance, with percentage production of fibre, DM, and NMC also being important traits.

The mean fibre production in the IGHs and ISHs was 16.45%, and ranged from 12.30% (99-438) to 21.15% (GU04-432). The best fibre producer amongst the standard commercial canes was CoS 767 (15.84%). Seven ISHs—2004-13 (19.77%), GU07-3730 (18.67%), KGS 2004-72 (18.15%), 99-488 (18.02%), GU 94-410 (17.78%), 2004-186 (17.66%) and GU 07-3784 (17.83%)—and three IGHs*,* namely, GU04-432 (21.15%), KGS 99-109 (19.68%), and KGS 99-104 (17.84%) had significantly higher fibre percentages as compared to the best commercial check. In overall experiment, ISHs group have recorded significantly higher biomass yield, number of stalks, and total dry matter percentage compared to other group whereas, IGHs group have recorded significantly higher fibre percent than ISHs (Table 3).

**Table 3:** Cane growth and quality traits with biomass-contributing traits of ISHs, IGHs, and commercial hybrids of sugarcane during Y1—Year 1 (2013–14) and Y2—Year 2 (2014–15).

S.no	Clones	SCW (kg)	CB%	JB%	NMC/ha	TDM%	Fibre %	FB (t/ha)
1	GU 94-410	1.58	12.02	18.53	59,279.18	29.49	17.78	108.17
2	99-81	1.11	8.19	17.05	83,813.11	30.17	16.93	103.9
3	99-132	0.82	10.19	18	78,282.83	29.26	16.19	92.62
4	99-438	1.28	8.16	17.6	76,941.88	28.8	12.30	120.18
5	99-488	0.9	8.89	18.7	78,787.88	31.83	18.02	104.19
6	99-489	1.1	7.82	18.4	96,459.75	23.77	16.07	118.15
7	KGS 2004-13	1.24	8.72	17.06	79,165.96	28.83	19.77	140.27
8	KGS 2004-48	2.25	10.48	18.63	70,664.79	20.97	14.36	157.46
9	KGS 2004-60	1.09	8.97	18.55	81,273.24	35.8	13.27	121.2
10	KGS 2004-72	1.16	10.14	18.8	52,273.94	30.32	18.15	84.7
11	2004-90	0.63	6.89	17.65	87,879.12	29.55	16.82	80.65
12	KGS 2004-186	1.74	10.65	18.38	55,560.03	27.79	17.66	141.27
13	GU 07-3704	0.85	4.65	13.35	138,172.05	22.78	14.18	165.96
14	GU 07-3730	0.43	5.98	17.3	124,982.26	40.42	18.67	85.86
15	GU 07-3764	0.91	7.46	18.45	102,525.2	32.53	16.57	101.69
16	GU 07-3784	0.65	5.51	16.9	120,212.02	37.35	17.83	95.46
17	GU 07-3849	0.87	4.94	15.41	158,593.01	28.34	17.28	138.52
18	GU 98-1395	1.09	10.53	17.35	62,622.15	29.71	17.25	85.16
19	KGS 99-100	1.39	7.89	16.8	90,326.65	24.11	16.85	163.99
20	KGS 99-104	1.14	9.52	18.15	60,479.55	29.98	17.84	79.68
21	KGS 99-109	0.78	8.16	16.46	106,565.66	28.18	19.68	97
22	GU 04-431	1.18	7.97	16.6	52,759.38	26.9	16.84	74.94
23	GU 04-432	1.99	9.87	16.53	59,854.42	19.78	21.15	125.47
24	Co 0238	1.49	11.71	20.25	80,303.54	24.85	13.05	118.82
25	CoJ 64	1.34	8.9	18.88	55,176.72	26.01	13.51	69.57
26	CoS 767	1.17	9.89	18.65	71,111.11	28.18	15.84	94.43
27	CoS 8436	1.24	10.56	19.03	70,110.45	23.24	15.1	103.62
28	1148-54-242-2	1.15	8.69	16.85	112,121.21	25.22	13.77	135.65
	Mean of ISH	1.09	8.22	17.57	90,874.49	29.88	16.58	115.31
	Mean of IGH	1.26	8.99	16.98	72,101.3	26.44	18.27	104.37
	Mean of Comm. vars	1.28	9.95	18.73	77,764.61	25.5	14.25	104.25
	Mean of the experiment	1.16	8.69	17.65	84,510.61	28.36	16.45	111.02
	Year	0.03	0.234	0.34	72,101.3	0.85	0.48	2.1
	Var	0.12	0.875	1.28	13,101.2	3.19	1.81	8.016
	Year × Var	0.17	1.237	1.8	18,528	4.51	2.56	11.33

*SCW* single cane weight, *CB* brix% in cane, *JB* juice brix%,* NMC* number of millable canes, *TDM* total dry matter, *FB* fresh biomass (t/ha^−1^), *Comm. vars* commercial varieties.

### Cane growth and quality parameters

None of the IGHs and ISHs performed better than the commercial cultivars with respect to juice quality traits such as Brix% in juice, sucrose% in juice and purity% (Table 3). However, six ISHs—KGS 2004-72 (18.80%), 99-488 (18.70%), KGS 2004-48 (18.63%), KGS 2004-60 (18.55%), GU 07-3764 (18.5%) and GU 94-410 (18.53%) were at par with regard to juice quality (Brix% in juice) with the best commercial standard cultivar Co 0238 (20.25%) at 10^th^-month crop stage. Among these hybrids, two ISHs*,* 99-488 and KGS 2004-60 had 18.70% and 18.55% juice brix, and 31.25 t ha^−1^, 32.93 t ha^−1^ dry biomass yield, respectively; therefore, these can be used as dual-purpose crops for sugar as well as biofuel production^29^. The overall mean for numbers of stalks per ha was significantly higher in one IGH, KGS 99-109 (1.07 lakh ha^−1^), and five ISHs—GU07-3849 (1.59 lakh ha^−1^), GU07-3704 (1.38 lakh ha^−1^), GU07-3730 (1.25 lakh ha^−1^), GU07-3764 (1.03 lakh ha^−1^), GU07-3784 (1.20 lakh ha^−1^), as compared to the population mean (0.85 lakh ha^−1^) and that of the best commercial cultivar (0.80 lakh ha^−1^). Single cane weight (SCW) of three ISH clones—GU 94-410 (1.58 kg), KGS 2004-48 (2.25 kg), and KGS 2004–186 (1.74 kg)—and two IGH clones, namely, KGS 99-100 (1.39 kg) and GU 04-432 (1.99 kg), was significantly higher than the population mean (1.16 kg).

### Winter sprouting index (WSI)

Three ISHs and one IGHs had significantly higher WSIs than the population mean and that of the best commercial check CoS 767. Out of these, one IGH clone, KGS 99-109, had the highest WSI of 4.52. Five ISHs—GU 07-3704 (3.43), GU 07-3730 (3.71), GU 07-3764 (2.3), GU 07-3784 (2.7), and GU 07-3849 (2.3) had significantly higher WSIs than the best standard CoS 767 (1.47) for WSI (see Supplementary Fig. S1).

### Energy value

Based on the analysis of means (ANOM), the clones 99-489, KGS 2004-13, KGS 2004-60, KGS 2004-186, GU 07-3704, KGS 99-100, and GU 04-432 were identified as having significantly higher energy potentials than the overall mean (461.61 Gj/ha/year) and the upper decision line (514.1 Gj/ha/year). The clones KGS 2004-72, 2004-90, GU 07-3730, GU 98-1395, KGS 99-104, GU 04-431, CoJ 64 and CoS 767 recorded significantly lower energy potentials than the overall mean and lower decision line. However, ten ISH clones, one IGH clone and three commercial canes have energy potentials at par with the population mean. Hence, these mentioned hybrids clones might be a good source of potential biomass energy^30,31^ (see Supplementary Fig. S2).

### Resistance to insects and pests

All the ISH and IGH clones of sugarcane with four standard varieties of cane were evaluated to observe their resistance against major insect pests, namely, the early shoot borer (ESB), top borer (TB), stalk borer (SB), and root borer (RB). All the clones were least susceptible (LS) to ESB and TB (< 15% and 10%, respectively). Twenty-three clones were found to be LS (infestation index < 2), while one clone, GU 07–3730, was moderately susceptible (MS) to SB (infestation index = 2.1–5.00). In case of the RB, 22 clones were LS (< 15%) and two clones were MS (15.1–30%) to the RB (Table 4).

**Table 4:** Evaluation of ISH/IGH clones for red rot and insect-pests.

S. No.	Clone name	Red rot reaction	Incidence (%)			Infestation index
			Early shoot borer	Top borer	Root borer	Stalk borer
1	GU 092-410	S	5.2	4.2	11.2	1.2
2	99-81	R	2.1	6.2	8.4	1.5
3	99-132	R	3.5	4.5	8.6	1.1
4	99-438	R	2.3	2.5	7.4	0.9
5	99-488	R	8.2	6.1	10.0	1.3
6	99-489	MR	1.6	2.0	3.1	1.4
7	KGS 2004-13	R	4.1	5.4	7.0	1.5
8	KGS 2004-48	R	2.1	5.2	2.1	1.3
9	KGS 2004-60	R	1.0	0	6.1	1.2
10	KGS 2004-72	R	7.1	4.5	9.1	1.1
11	KGS 2004-90	MS	5.6	6.1	12.0	1.0
12	KGS 2004-186	R	6.4	5.9	16.9	1.7
13	GU 07-3704	HS	2.2	7.1	2.0	1.2
14	GU 07-3730	MS	6.3	3.4	4.8	2.9
15	GU 07-3764	MR	4.1	4.6	7.1	1.0
16	GU 07-3784	HS	9.2	8.1	2.1	1.6
17	GU 07-3849	MR	7.5	2.1	4.1	1.7
18	GU 98-1395	MR	1.1	1.8	15.2	2.1
19	KGS 99-100	MR	2.4	4.1	4.1	1.3
20	KGS 99-104	MR	6.2	6.9	6.1	1.5
21	KGS 99-109	MR	1.7	6.1	7.5	1.2
22	GU 04-431	R	5.5	7.1	6.2	1.8
23	GU 04-432	MR	5.9	6.1	3.5	1.2
24	Co 0238	MR	1.4	7.4	7.1	1.9
25	CoJ 64	S	2.5	6.9	8.1	1.6
26	CoS 767	MS	7.4	6.1	7.1	1.2
27	CoS 8436	MS	2.1	7.0	6.1	1.1
28	1148-S4-242-2	R	7.5	3.1	8.1	1.0

### Resistance to red rot

The resistance of ISHs and IGHs to red rot were recorded using mixed inocula of two virulent races of *C. falcatum* (CF08 and CF09) prevalent in subtropical India. Out of seventeen ISHs screened, three clones exhibited resistance (R), nine were moderately resistant (MR), two were moderately susceptible (MS), and three were susceptible (S) to this disease (Table 4). The ISH clones 99-81, 99-132, 99-438, and 99-488 derived from population improved *officinarum* (PIO) clones, and the ISH clones KGS 2004-13, KGS 2004-48, KGS 2004-60 and KGS 2004-72, derived from *S.* *barberi* and *S.* *sinense *were rated as resistant. In addition, two ISH clones (GU 07-3764 and GU 07-3849) derived from *S. officinarum* and *S. spontaneum* backgrounds were rated resistant to red rot. All the six IGHs were either resistant or moderately resistant to red rot. Out of these, four IGHs, namely, GU98-1395, KGS 99-100, KGS 99-104, and KGS 99-109 were derived from commercial cane crossed with *E. arundinaceus* and two IGHs—GU04-431 and GU 04-432—were derived from *S. robustum* and *E. arundinaceus*.

### Cluster analysis

**Figure 2:**
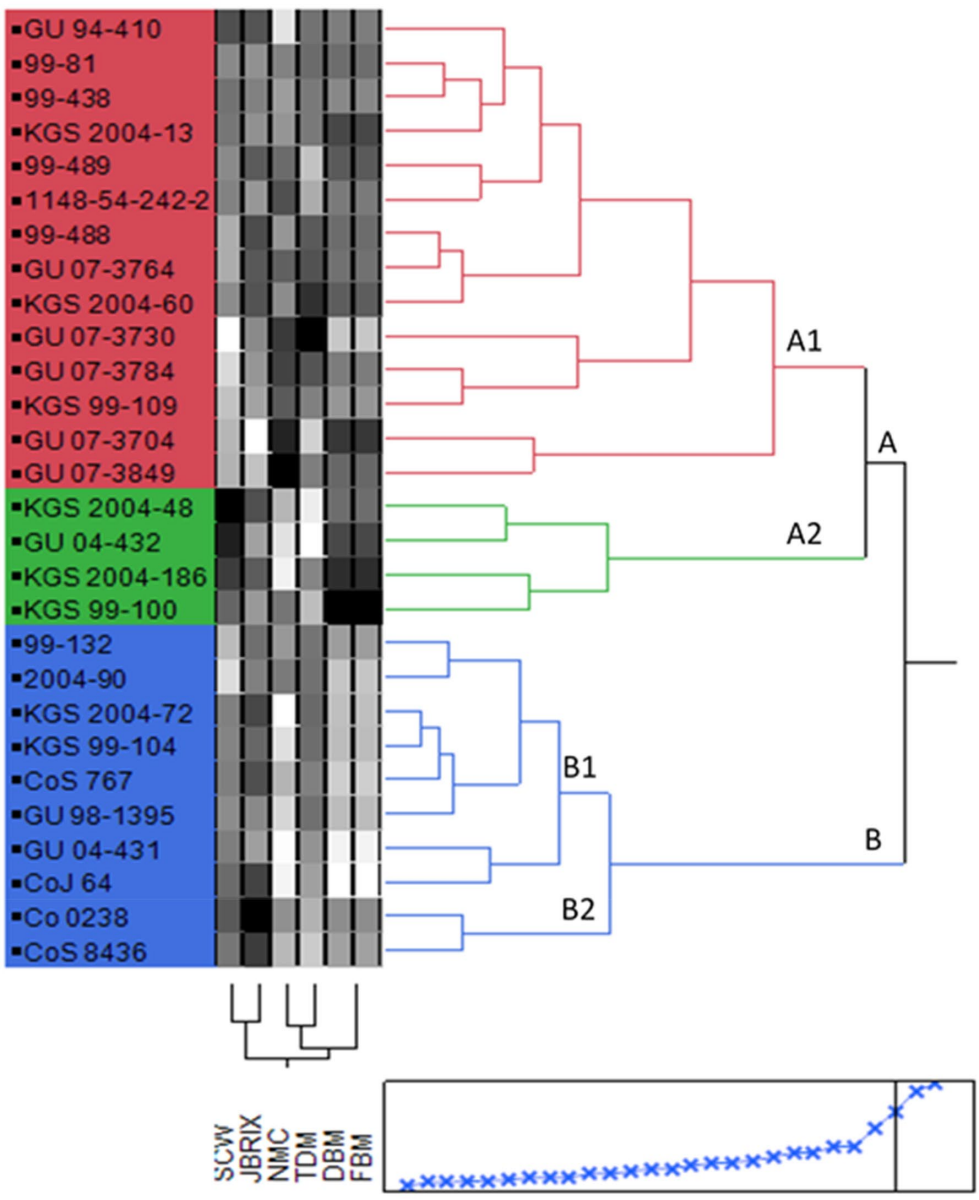
Two-way hierarchical clustering of 28 sugarcane hybrids clones. *SCW* single cane weight, *JBRIX* juice brix, *NMC* number of millable canes, *TDM* total dry matter, *DBM* dry biomass matter, *FB* fresh biomass matter.

Two-way cluster analysis separated the ISH and IGH clones into two major groups based on their traits (Fig. 2). Group A contains 18 hybrid clones, of which 10 are ISHs (GU 09-410, 99-438, 99-488, 99-489, 99-81, GU 07-3704, GU 07-3730, GU07-3764, GU07-3784, and GU 07-3849) derived from *S. officinarum, S. spontaneum,* and *S. robustum* backgrounds. Four of the ISHs (KGS 2004-13, KGS 2004-186, KGS 2004-48, and KGS2004-60) were derived from *S. barberi* and *S. sinense* backgrounds. One hybrid was a selfed progeny of cultivar Co 1148. In addition, three hybrids (KGS 99-100, KGS 99-109, and GU04-432) were derived from the intergeneric background of *E. arundinaceus* with commercial cane*.*

Group B contains ten hybrids of diverse origins. This group is sub-divided into two subgroups (B_1_ and B_2_) representing eight and two hybrids, respectively. Subgroup B_1_ contains two hybrids from the *S. barberi* and *S. sinense* backgrounds and two hybrids from the intergeneric background of *E. arundinaceus.* Subgroup B_2_ contains commercial hybrids of sugarcane, and subgroup B_1_ contains one hybrid from S*. officinarum*, *S. spontaneum,* and *S. robustum* backgrounds.

## Discussion

The genetic variation found in *Saccharum* germplasm may play a very significant role in improving sugarcane for biomass production through breeding and biotechnological approaches. Sugarcane intergeneric and interspecific species like *Miscanthus, Erianthus*, *S. officinarum*, and *S. spontaneum* have a lot of allelic diversity and can be exploited further for improving sugarcane biomass yield^17,32,33^. It was possible to develop high dry biomass hybrid clones when the existing commercial cultivars are combined with high biomass type clone^22,34,35^.

Miocque (1999)^34^, who investigated sugarcane growth and biomass for ten crops in Sao Paulo State (Brazil) reports that the highest biomass accumulation occurs when sugarcane has a longer growth period. Further, the potential for crude energy production of sugarcane clones can be enhanced by using fibre-rich species. One of the important findings of this study is that the early generation hybrids of *S. spontaneum* (F_1_ and BC_1_) and *E. arundinaceus* (F_1_) had higher biomass yields than the later generation clones. This indicates a limited variation in modern cultivars (commercial canes) and necessitates the utilization of wild relatives in improving biomass in sugarcane (Table 3).

Two-way analysis broadly separated the accessions based on trait variation. Group A contains sugarcane hybrids with higher single cane weights, juice brix percentages, and numbers of stalks. This group contains mostly commercial cultivars and other hybrids of diverse origin.

The commercial cultivars Co 0238, CoJ 64, and the interspecific hybrids KGS 2004-48, KGS 2004-186, KGS 2004-60, and GU07-3764 can be used as donors for improving quality traits in sugarcane. The interspecific hybrids GU 07-3847, GU 07-3704, GU 07-3730, and GU 07-3784 can be used further as donor parents for improving yield traits. Group B had higher fresh biomass, total dry matter percentages, and dry biomass-related traits. The IGH KGS 99-100 and ISHs GU 07-3704, KGS 2004-13, KGS 2004-186, KGS 2004-60 and 99-489 can be used as good sources for high biomass producing hybrids for the Indian subtropics. The clustering of trait variations among the ISHs and IGHs can allow breeders to choose different traits from the different clusters for crossing programs. It is also indicated that these potential performers may be considered as candidate donors for further exploiting traits variation for genetic improvement of the sugarcane.

Since trait improvement in sugarcane through marker assisted selection (MAS) and genomic selection at the molecular level is difficult due to its genomic complexity (large genome size and high polyploidy level)^36–38^, efforts were made in this study to introgress genes for improvement of biomass yield from allied genera and other species of *Saccharum.* Out of 28 hybrid clones that were evaluated in this study, five clones of ISHs—2004-186, GU 07-3704, KGS 2004-13, KGS 2004-60 and 99-489—showed significantly higher dry biomass yields as compared to the population mean indicating their superiority over the existing commercial cultivars. Since the ISHs have *S. officinarum, S. spontaneum, S. robustum, S. barberi,* or *S. sinense* as one of the parents in their backgrounds, it is likely that these species have contributed traits that have significantly improved morphology, good ratooning, and tolerance to various biotic and abiotic stresses^39,40^. In addition, since these species are ecologically well adapted to subtropical Indian conditions, their hybrid clones can grow well even in marginal lands. ISHs—GU 07-3704, GU 07-3730, GU 07-3764, GU 07-3784, and GU 07-3849—showed significantly superior winter sprouting over the best standard CoS 767 (supplementary Fig. S1), because of these hybrids background contains contribution of the genome from population improved *S. officinarum* and *S. spontaneum*. Screening against red rot from the wild germplasm of sugarcane, allows new sources of resistant and red rot resistant can be transferred into cultivated species through interspecific and intergeneric crosses^41^. In the study, ISHs clones-derived from population improved *officinarum*, *S. barberi* x *S. sinense* and *S. officinarum* × *S. spontaneum,* were reported as resistant against red rot. Among the species, *S. spontaneum* originated clones have high level of resistance against red rot^42^, and effectiveness of *S. spontaneum* in sugarcane breeding had been evident since the development of first interspecific hybrid ‘Co 205’ in sugarcane. IGH clones—derived form *E. arundinaceous* with commercial cane and *S. robustum* with *E. arundinaceous* produced resistant clones against red rot. Use of *Erianthus spp*. as potential source for diversifying the genetic base and red rot resistance in sugarcane^43^. Furthermore, the majority of clones studied were least susceptible for early shoot and root borers due to their wider genetic makeup, hence showed resistance reactions against the major insets prevailing in subtropics.

Our results show that ISH and IGH clones performed well in the field conditions. Sugarcanes, *Miscanthus* species (*Miscanthus giganteus*), *Erianthus* species (*E. arundinaceus* Retz.), and switchgrass (*Panicum virgatum*) are known to be efficient biomass accumulators and converters of solar energy into chemical energy^11,44^. These traits were also found in the IGHs KGS 99-100 and GU 04-432, which were significantly better than the best commercial cultivar Co 0238 in producing dry biomass. Similarly, in terms of estimated energy, IGH clone KGS 99-100—a hybrid derivatives of the commercial cane and *E. arundinaceous*, and clone GU04-432—a hybrid derivatives of *S. robustum* and *E. arundinaceous*, had significantly higher energy value than the existing commercial varieties (supplementary Fig. S2). Therefore, these hybrids could be good sources for improving biomass and bioenergy traits in popular cultivars of sugarcane*. Erianthus spp*. due its higher biomass accumulation and calorific value, good ratoon ability and exceptional adaptability to biotic and abiotic stresses, is considered as potential breeding material for use in future sugarcane breeding programs^45^. Intergeneric material is powerful tools for widening the genetic base in polyploidy crop breeding. Harvey et al. (1994)^46^ also suggested the use of *Miscanthus* and *Erianthus* species for broadening genetic bases in sugarcane breeding programs. Furthermore, the use of ISH and IGH clones in sugarcane hybridization can tap into many relevant genetic variations in the germplasm for better biomass production traits while also broadening the genetic base^47^.

Though biomass improvement in sugarcane can be challenging due to several issues such as the small gene pool in currently available sugarcane cultivars, long breeding periods and selection cycles, poor synchronization and fertility of flowers in parental lines, and genome complexity associated with this^48,49^. Since wild sugarcane species have more genetic variability than domesticated sugarcane cultivars, it makes sense to use ISHs and IGHs clones to improve biomass traits in sugarcane. In addition, breeding efficiencies can be improved by selecting for parental strains with wide genetic backgrounds coupled with introgression of genes for improving biotic and abiotic stress tolerance.

In conclusion, our **s**tudy shows that the utilization of the *Saccharum* and *Erianthus* species in sugarcane breeding programs has the potential for generating clones with enhanced biomass accumulation and yields. The tremendous genetic diversity present in these species could be used as a significant genetic reservoir for improving biomass and bioenergy production traits in commercial canes which have narrow gene pools. Currently, long breeding cycles, non-synchronous flowering, and poor fertility among the desired parents are major bottlenecks for the efficient improvement of sugarcane for biomass contributing traits. These hurdle’s can be overcomes by utilizing ISH and IGH clones in sugarcane breeding programs to develop superior clones of high biomass potential and high tolerance for abiotic and biotic stresses. Generated clones with increased biomass and bioenergy traits in this study will be good genetic material for further utilization in sugarcane breeding programs in improving biomass-energy traits. Furthermore, these clones could be grown in lands other than prime agricultural land (thereby avoiding competition for land with food industries) to meet the growing demands for energy.

## Methods

### Plant material and experimental site

All experimental were carried out on 28 clones obtained from 17 ISHs and six IGHs (IGH), one inbred, and four commercial sugarcane checks (two early—Co 0238, CoJ 64, and two mid–late—CoS 767, CoS 8436 checks (Table 1). These clones were evaluated for their biomass production potential and other related traits under subtropical conditions in India. The population-improved *S. officinarum* (PIO), *S. spontaneum* (SIP), and *S. robustum* (PIR) clones used in this study were the product of intra population improvement programs for their respective species.

**Figure 3:**
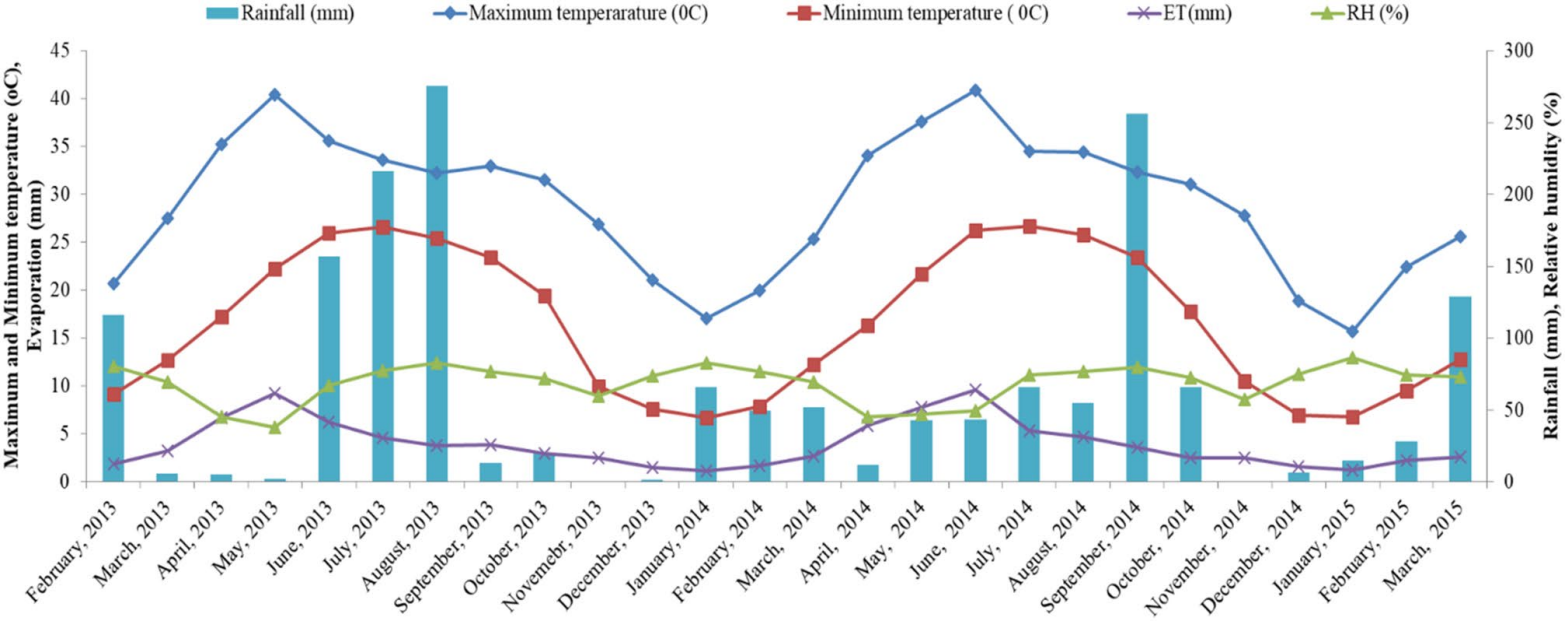
Maximum and minimum temperatures, evaporation rates, and relative humidities of Karnal (Haryana State, India) during the two-year experimentation period.

The experimental site was located at the ICAR-Sugarcane Breeding Institute, Regional Centre, Karnal (Haryana) India (29.1°–29.5° N and 76.3°–77.1° E), which has a subtropical climate. The location stands at an elevation of 243 m above mean sea level and receives an average rainfall of ~ 744 mm per year. The maximum temperatures here range from 34–45 °C in summer, and minimum temperatures range from 5–8 °C in winter. The maximum and minimum temperatures, evaporation rates, relative humidity, and rainfall during 2013–14 and 2014–15 are shown in Fig. 3. The soil in this area ranges from clay-loamy to loam, with a pH range from 8.0–8.5; the site is irrigated from a bore well source.

### Experimental design

The experiment was conducted in a randomized block design with three replicates for two years (during 2013–14 and 2014–15 spring seasons). The plots used were 2 m × 6 m with 0.9 m spacing in size. Recommended crop production practices were followed for raising the experimental crop. Data on the numbers of millable canes (NMC), brix% in cane, single cane weight (SCW), dry cane weight (DCW), total dry weight (TDM), and fresh biomass weight (FBW) were recorded for each plot from five randomly tagged canes at a crop age of 10 months. Five canes from each plot were used for estimating the percentage of fibre; a subsample of 250 g of the shredded canes were crushed in a rapipol machine and oven dried. The fresh weights and dry weights of these samples were recorded. Fibre percentage was estimated using the rapipol extraction method and calculated as per the method explained in Thangavelu and Rao (1982)^50^.

$${\text{Fibre percent}} = \frac{A - B}{C} \times 100$$

where A is the dry weight of bag + bagasse after drying (g), B is the dry weight of bag alone (g), C is the fresh weight of cane (g).

Juice brix and sucrose percentages were measured from extracted cane juice. Brix percentage in cane and dry cane weight were estimated in the 12th month of the crop, i.e., the harvest stage. Fresh biomass yield was worked out from single cane weight and NMC per plot. CCS percentage at the 12th month was computed as per the method explained in Chen and Chou (1993)^51^. To estimate the dry matter percentage, brix percentage in cane, and dry biomass yield, the following formulae were used.

$${\text{DM percent in cane }} = \frac{{\left( {WSB - WSA} \right) \times \, \frac{Brix \, in \, Juice}{{100}} + \, \frac{WSA \, \times DMB}{{100}}}}{WSB \, \times \, 100}$$

where WSB is the weight of the sample before crushing (g), WSA is the weight of the sample after crushing i.e. Bagasse (g) and DMB is the dry matter content in bagasse (%).

$${\text{Brix percent in cane }} = \frac{Juice extraction \% \times Juice Brix \% }{{100}}$$

Fresh biomass yield (t ha^−1^) was calculated from the number of millable canes/ha × weight of a single cane (kg) with top attached. Dry biomass yield was calculated in t ha^−1^ using following formula described by Mohanraj et al., 2014^21^.

$${\text{Dry Biomass yield }}\left( {{\text{t}}/{\text{ha}}} \right) \, = Dry\;matter\;\% \times Fresh\;biomass\;yield\;{(t/ha^{ - 1} )}$$

To identify clones with better ratoonability when harvested during the peak winter period in subtropical climates (i.e., January when temperatures fall below 5 °C), sprouting of ISH/IGH clones was quantified. This was done by harvesting the plant crop (half of the row/replicate (3 m row length), and counting the numbers of sprouted clumps per row and recording the average numbers of shoots/sprouted clumps in February; using these, the WSI was calculated^52^. The number of stubbles sprouted per plot and number of shoots formed per clump during winter was counted on the 45th day after ratooning. To ascertain winter sprouting potential of sugarcane clones, a winter sprouting index (WSI) was used as per Bakshi Ram et al. (2017)^52^; the WSI is calculated as follows:

$${\text{WSI}} = \frac{{\left( {{\text{\% of Sprouted clumps per plot}}} \right){ } \times { }\left( {\text{number of shoots per clumps}} \right)}}{100}$$

### Red rot screening

To identify their resistance to red rot, all ISH and IGH clones were screened against the most prevalent and virulent pathotype of red rot (C*olletotricum falcatum*), namely, *Cf 08* and *Cf 09* under field conditions. A mixed inoculum of both races was inoculated into sugarcane plants by plug and nodal methods during the month of September in the 7-month old crops^53^.

### Energy calculation

The energy content of plant mass mainly depends on its composition; fats and proteins have higher energy contents than simple carbohydrates. Sugarcane is mainly composed of carbohydrates (sugar and lignocellulose) that have an energy content of (~ 15.9 MJ/kg)^54^. Energy content was calculated by multiplying the total dry biomass by 15.9 and expressed as Gigajoule/ha/year (Gj/ha/year).

### Statistical analysis

All data were subjected to analysis of variance (ANOVA) tests. Means, standard deviations, and coefficients of variance for different traits were computed using the statistical package SAS 9.3 software (SAS Institute Inc., Cary, USA). Cluster analysis was performed using the JMP pro 10.0 version to elucidate the differences between IGHs, ISHs, and commercial varieties of sugarcane. Analysis of means (ANOM) was done for comparing energy values and winter sprouting index (WSI) values of the different ISHs, IGHs, and commercial sugarcanes.

## Supplementary Information

Supplementary Figure S1.

Supplementary Figure S2.

## Data Availability

The data generated during and/ or analyzed during the current study are available from the corresponding author on reasonable request.
