# Supplementary material for: Biomass potential of novel interspecific and intergeneric hybrids of Saccharum grown in sub-tropical climates
Source: Sci Rep. 2020 Dec 9;10:21560. doi: 10.1038/s41598-020-78329-8 (PMC7726553; doi:10.1038/s41598-020-78329-8)
Supplement: Supplementary file 2 — Supplementary Figure S2. [file 41598_2020_78329_MOESM2_ESM.pdf]

Biomass potential of novel interspecific and intergeneric hybrids of *Saccharum* grown in sub-tropical climates

Mintu Ram Meena*[1], Ravinder Kumar[1], Karuppaiyan Ramaiyan [1,2], Manoharlal Chhabra[1], Arun Kumar Raja [2], Mohanraj Krishnasamy [2], Neeraj Kulshreshtha[1], Shashikant Pandey[1] and Bakshi Ram[1,2]
[1]ICAR- Sugarcane Breeding Institute, Regional Centre, Karnal, India
[2]ICAR-Sugarcane Breeding Institute, Coimbatore, India
*Corresponding author Email: mintu_uas@yahoo.co.in

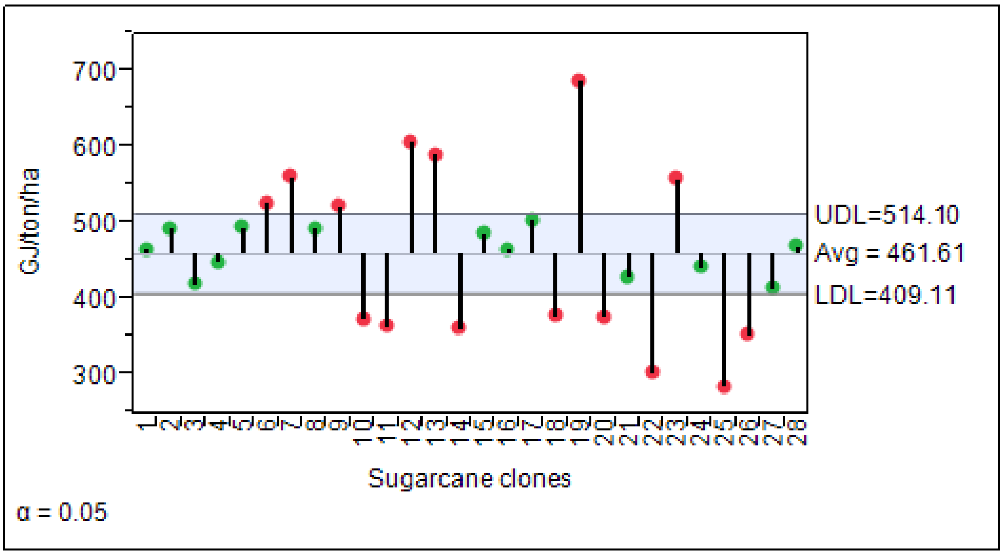

Supplementary Fig. S2**.** Energy value of interspecific (ISH), intergeneric (IGH), and commercial hybrids of sugarcane. UDL: upper decision line, LDL: lower decision line, and Avg: average.
